# Stereoselective cathodic synthesis of 8-substituted (1*R*,3*R*,4*S*)-menthylamines

**DOI:** 10.3762/bjoc.11.34

**Published:** 2015-02-27

**Authors:** Carolin Edinger, Jörn Kulisch, Siegfried R Waldvogel

**Affiliations:** 1 Institut für Organische Chemie, Johannes Gutenberg-Universität Mainz, Duesbergweg 10-14, 55128 Mainz, Germany

**Keywords:** cathodic reduction, chiral amines, electrosynthesis, menthylamines, oximes

## Abstract

The electrochemical generation of menthylamines from the corresponding menthone oximes equipped with an additional substituent in position 8 is described. Due to 1,3-diaxial interactions a pronounced diastereoselectivity for the menthylamines is found.

## Introduction

Optically active amines serve as powerful and versatile tools in organic synthesis. Among numerous applications they are applied as chiral ligands [1], as catalysts for various asymmetric transformations [2], and as building blocks for alkaloid and pharmaceutical drug synthesis [3–4]. The increasing number of applications leads to a growing interest in the stereoselective preparation of such amines. Throughout the last decades, several strategies [3] such as stereospecific amination via C–H insertion [5–8] or asymmetric olefin hydroamination [9–13] have been investigated in order to obtain access to optically pure amines. However, the major fraction of starting materials for the synthesis of these compounds is still provided by the chiral pool. Usually, optically active alcohols or amino acids serve as starting material for such amine syntheses [14]. Naturally occurring terpenes such as carene [15], limonene [16], pinene [17–18] or camphor [19] are used as precursors for chiral β-amino alcohols. As precursors for α-chiral primary amines, fenchone [20] and camphor [21–22] are typically employed. Furthermore, optically pure dehydroabietylamine is readily available and applicable without further modification [23–25].

**Scheme 1:**
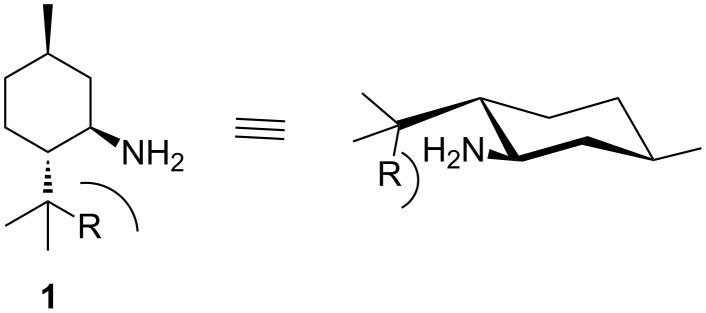
Structural features of 8-substituted menthylamines **1**.

Among the terpenoid derived amines, menthylamine and its 8-substituted derivatives **1** represent a particularly interesting candidate. Due to the strong steric influence in the vicinity of the amino functionality, high selectivity can be expected for asymmetric transformations [26–27], since appropriate moieties as substituents R create a molecular U-turn (see Scheme 1).

**Scheme 2:**
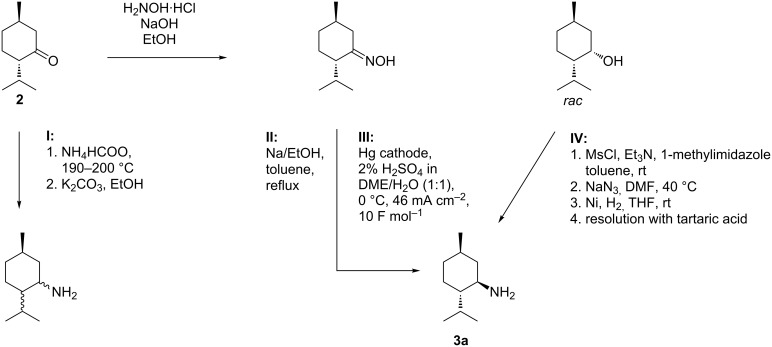
Synthetic strategies to menthylamines.

However, while optically pure menthol derivatives play a significant role in organic synthesis [28], menthylamines have only been used in a few situations, which is attributable to their rather poor availability in optically pure manner. Among the applications reported so far, are the uses as building blocks in supramolecular receptors [29–34] or the synthesis of high-performance stationary phases for liquid chromatography [35–38]. (−)-Menthone (**2**) can be converted to menthylamine by different methods: A general way to convert naturally occurring terpenoids is the reductive amination under Leuckart–Wallach conditions (see Scheme 2, pathway **I**) [39]. This method was applied to convert **2** to *N-*alkyl substituted menthylamines [40]. However, a significant disadvantage of this method is the lack of stereocontrol and partial inversion of the configuration at position 4 resulting in a complex diastereomeric mixture. Alternatively, reduction of menthone oxime can be achieved, either employing Bouveault–Blanc conditions [41], or via hydrogenolysis at a transition metal catalyst [42]. Both approaches lead to the desired product as a diastereomeric mixture.

In the past, we elaborated straightforward and efficient approaches to optically pure (−)-menthylamine (**3**, see Scheme 2, pathways **II**, **III**, **IV**).

**Scheme 3:**
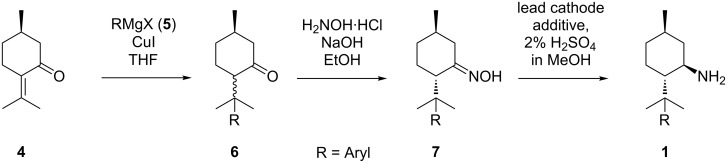
Stereoselective synthesis of 8-substituted (1*R*,3*R*,4*S*)-menthylamines.

One protocol involves conversion of the inexpensive technical intermediate racemic neomenthol to menthylamine in a three-step sequence, followed by enantiomeric resolution employing tartaric acid (see Scheme 2, pathway **IV**) [43]. By the amount of water in the crystallisation mixture the precipitation of the desired diastereomeric salt can be chosen [44]. Furthermore, we developed a Bouveault–Blanc-type protocol where (−)-menthone oxime is converted to **3** in attractive diastereoselectivity (see Scheme 2, pathway **II**) [45]. However, the necessity for excess amounts of sodium metal constitutes a major drawback. Consequently, we also investigated on electrochemical alternatives for the reduction of (−)-menthone oxime. In this context, we found that it can be efficiently converted to the corresponding diastereomeric amines with an excess of **3a** in a divided cell under galvanostatic conditions employing an Hg pool cathode (see Scheme 2, pathway **III**) [26]. Here, we report a new synthetic route to optically pure 8-substituted menthylamines **1** starting from commercially available (+)-pulegone (**4**, Scheme 3). An important feature of this sequence is the initial introduction of a sterically demanding moiety R in position 8 via cuprate addition. After conversion to menthone oximes **7** within the second step, R is supposed to enhance the stereoselectivity of the following electrochemical reduction process (Scheme 3, step 3). Concomitantly, the resulting amines **1** are expected to have improved properties as catalysts/auxiliaries for asymmetric transformations due to the increased sterical demand in the vicinity of the amine group.

## Results and Discussion

### Exploratory work

**Scheme 4:**
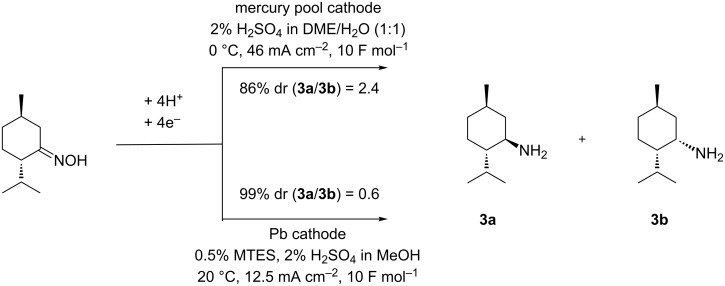
Influence of the cathode system onto the stereoselectivity of the reduction of (1*R*,4*S*)-menthone oxime.

Our previous studies revealed that menthone oxime can be electrochemically reduced on either mercury pool or lead cathode (see Scheme 4) [26]. Under optimized reaction conditions, the use of a mercury pool cathode renders compound **3** in 86% yield with a diastereomeric ratio (dr) **3a**:**3b** of 2.4. In contrast, using a lead cathode under optimized conditions leads to a reversed diastereomeric ratio of 0.6 in 99% yield. Notably, methyltriethylammonium methylsulfate (MTES) was used as additive in the latter case in order to suppress lead corrosion and to improve the current efficiency [26,46–47]. These promising results prompted us to study the stereoselectivity of the reduction in the presence of a sterically demanding group in position 8 of the substrate molecule.

### Synthesis of the menthone oxime substrates

**Scheme 5:**
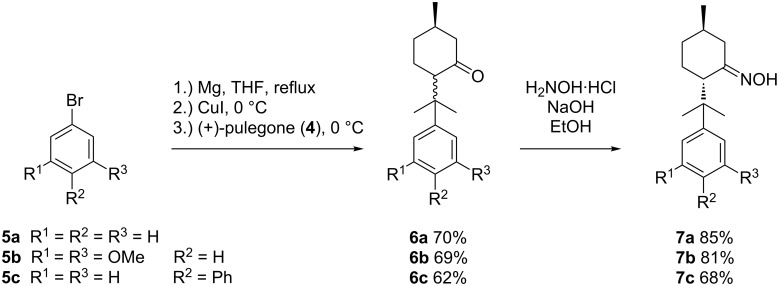
Preparation of 8-substituted (1*R*)-menthones **6** and the corresponding oximes **7**.

In order to obtain the desired 8-substituted (1*R*,4*S*)-menthone oxime substrates **7** we started from commercially available (+)-pulegone (**4**) in technical grade (92%) (see Scheme 5). First, the desired aryl moiety was installed by cuprate addition generated from aryl bromide **5**, yielding the corresponding menthones **6a**–**c** in good yields. In all cases, simple distillation is sufficient to isolate **6** in high purity.

Subsequent treatment of epimeric mixtures **6a**–**c** with NH_2_OH∙HCl and NaOH provides the corresponding (1*R*,4*S*)-menthone oximes in good yields (68–85%). For both, cuprate addition and oxime formation, conversions of the compounds exhibiting the diphenyl moiety in position 8 (**6c** and **7c**, R^2^ = Ph) require prolonged reaction times and render slightly lower yields.

### Electrochemical reduction of 8-substituted menthone oximes

**Scheme 6:**
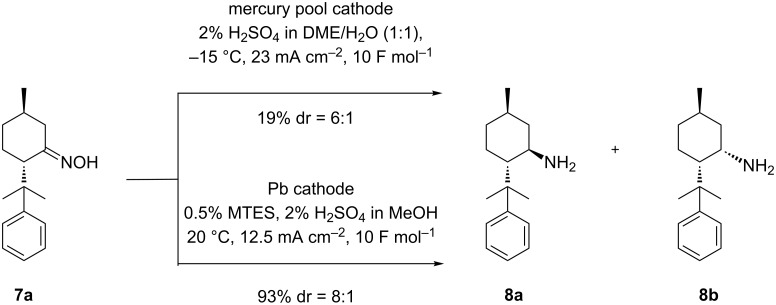
Influence of cathode material on the preparation of (1*R*,3*R*,4*S*)-menthylamine **8a**.

The electrolyses were carried out in a divided cell using a Nafion^®^ sheet as separator and platinum as anodic material. Since previous studies revealed that the reduction of the non-substituted menthone oxime was most effective on mercury or lead as cathodic material [26], we were prompted to study the influence of such cathodes onto yield and stereoselectivity in the conversion of **7a** (see Scheme 6).

The results clearly indicate that lead represents the superior cathode for this application. Compared to mercury, the use of a lead cathode renders significantly higher yields (93% vs 19%). Furthermore, an improved dr of 8:1 in favor of the desired isomer **8a** is obtained (compare dr = 6:1 obtained at Hg). Both results clearly demonstrate the positive effect of the substituent in position 8 onto the diastereoselectivity of the reduction (compare Scheme 4, dr = 2.4). Since lead was distinctly the prime cathode material it was used throughout all further investigations. To determine the influence by the temperature onto the selectivity, the reaction was carried out at 20, 40, and 60 °C (see Table 1). Methyltriethylammonium methylsulfate (MTES) serves as additive in order to suppress electrode corrosion due to PbSO_4_ formation [26,46–47].

**Table 1:** Effect of temperature onto yield and stereoselectivity of the electrochemical reduction of **7a** on a lead cathode.

Entry^a^	*T* [°C]	yield^b^ [%]	c.e.^c^ [%]	dr^d^ [**8a**:**8b**]

1	60	33	13	6.2
2	40	85	34	5.4
3	20	93	38	5.2

^a^Catholyte: 0.5% MTES and 2% H_2_SO_4_ in MeOH, current density: 12.5 mA cm^–2^, passed charge: 10 F mol^–1^, anode: platinum; ^b^yield determined by GC (internal standard); ^c^c.e. = current efficiency; ^d^ratio determined by NMR spectroscopy.

With increasing temperature higher diastereoselectivity can be observed, but the product yield strongly decreases. At 60 °C, hydrolysis of the oxime becomes the predominant reaction, yielding large amounts of menthone **6a** and the desired product **8** in only 33% yield (Table 1, entry 1). Moreover, the influence of alkylammonium salt additives on the reaction was studied (see Table 2). Such additives are known to have a positive effect on reductions on lead cathodes due to the formation of an ionic coating on the electrode surface and concomitant inhibition of cathodic corrosion and evolution of molecular hydrogen [46–47].

**Table 2:** Influence of alkylammonium salts on the electrochemical preparation of **8a**.

Entry^a^	additive	yield^b^ [%]	c.e.^c^ [%]	dr^d^ [**8a**:**8b**]

1	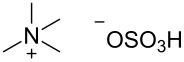 **9**	83	33	7.5
2	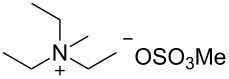 **10**	93	37	6.2
3	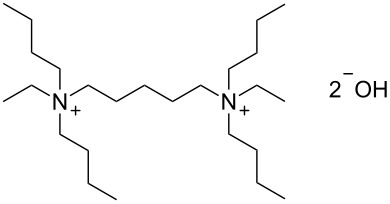 **11**	95	38	4.7

^a^Catholyte: 0.5% additive and 2% H_2_SO_4_ in MeOH, current density: 12.5 mA cm^−2^, passed charge: 10 F mol^−1^, anode: platinum; ^b^yield determined by GC (internal standard); ^c^c.e. = current efficiency; ^d^ratio determined by NMR spectroscopy.

The chosen additives differ in size and number of ammonium groups. The use of additive **11** leads to the highest product yield (Table 2, entry 3) but with a dr of 4.7 the lowest stereoselectivity. However, optimal compromise is given by MTES (additive **10**) as additive with a yield of 93% and a diastereomeric ratio of 6.2 (Table 2, entry 2). The compact cation **9** renders the best result with regard to diastereomeric ratio, but with inferior yield of 83%.

Next, the elaborated conditions were applied to oxime **7b** (Table 3, entry 2). The corresponding menthylamines **12a** and **12b** were obtained in only 42% yield in a diastereomeric ratio of 6:1. However, the yield can be significantly improved using other alkylammonium additives, such as tetramethylammonium salt **9**, BQAOH (**11**) and cyclic alkylammonium salt **13** (see Table 3, entries 1, 3 and 4), whereas **10** and **13** render improved yields along with lower diastereoselectivity, the use of additive **11** provides the highest yield with best stereoselectivity (see Table 3, entry 3). Again, the positive effect of the substituent in position 8 onto the diastereoselectivity of the reduction is clearly demonstrated (compare Scheme 4).

**Table 3:** Electrochemical synthesis of **12a** using different additives.

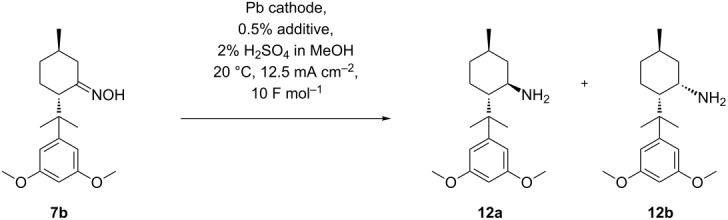				

Entry^a^	additive	yield^b^ [%]	c.e.^c^ [%]	dr^d^ [**12a**:**12b**]

1	**9**	63	25	6.8
2	**10**	42	17	5.6
3	**11**	78	31	8.6
4	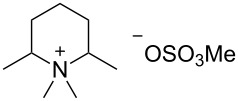 **13**	73	29	4.4

^a^Anode: platinum; ^b^yield determined by GC (internal standard); ^c^c.e. = current efficiency; ^d^ratio determined by NMR spectroscopy.

Since the achieved results with additive **11** are very promising (78%, dr 8.6) we were prompted to perform further studies on the effect of the concentration of **11** contained in the electrolyte. The results reveal that a concentration of 1 wt % renders optimum values for product yield, current efficiency and diastereomeric ratio (see Table 4).

**Table 4:** Influence of the concentration of additive **11** on the electroreduction of **7b**.

Entry^a^	*c* (additive **11**) [%]	yield^b^ [%]	c.e.^c^ [%]	dr^d^ [**12a**:**12b**]

1	0.5	78	13	8.6
2	1	89	36	8.9
3	4	85	37	7.8

^a^Catholyte: 2% H_2_SO_4_ in MeOH, current density: 12.5 mA cm^−2^, passed charge: 10 F mol^−1^, anode: platinum; ^b^yield determined by GC (internal standard); ^c^c.e. = current efficiency; ^d^ratio determined by NMR spectroscopy.

**Scheme 7:**
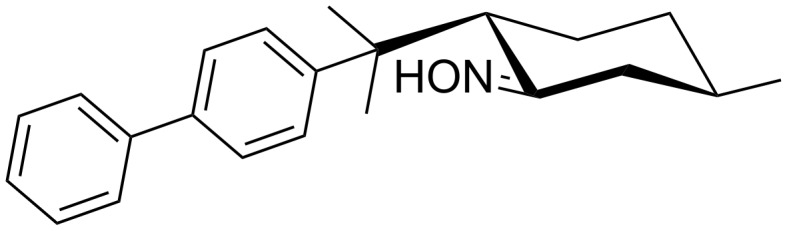
Protection of the oxime functionality in **7c** due to the sterically demanding diphenyl moiety in 8-position.

Increasing the additive amount to 4 wt % does not have any further benefit onto the oxime conversion, while simultaneously the stereoselectivity decreases slightly (Table 4, entry 3). Employing oxime **7c** demonstrates the limitations of our methodology. After passing 10 F under the optimized reaction conditions using additive **10**, non-converted oxime **7c** was fully recovered. A possible explanation is a strong decrease of the electron transfer rate due to steric shielding of the oxime functionality (see Scheme 7). Also the low solubility of the substrate is obstructive.

### Diastereomerically pure 8-substituted menthylamines

**Scheme 8:**
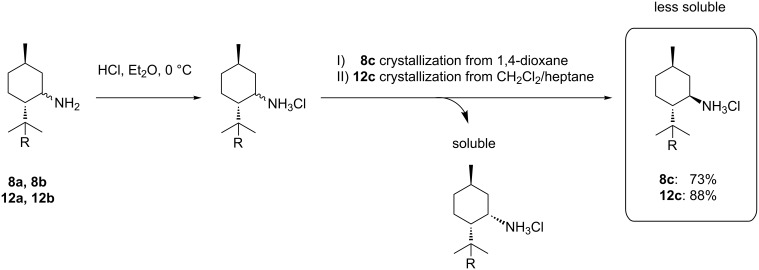
Separation of the diastereomeric 8-substituted menthylamines by crystallization of their hydrochlorides.

To obtain diastereomerically and analytically pure 8-substituted (1*R*,3*R*,4*S*)-menthylamines, each diastereomeric mixture can be transformed to the corresponding hydrochlorides by passing HCl through a solution of **8** or **12** in diethyl ether followed by a selective crystallization of diastereomers **8c** and **12c** from 1,4-dioxane or CH_2_Cl_2_/heptane, respectively (see Scheme 8). **8c** and **12c** can thus be obtained in 73% and 88% yield.

## Conclusion

We presented an efficient strategy to synthesize (1*R*,3*R*,4*S*)-menthylamines with aryl substituents in position 8. Starting from commercially available (+)-pulegone, the desired aryl moiety can be installed in position 8 by cuprate addition. Subsequent treatment with NH_3_OH∙HCl at alkaline conditions provides the enantiomerically pure (1*R*,4*S*)-menthone oximes in good yields. In the final step of the reaction sequence, the oxime is electrochemically reduced. We found that the use of a lead cathode in combination with alkylammonium additives render the desired 8-substituted (1*R*,3*R*,4*S*)-menthylamines in high yields and good diastereoselectivity. Compared to non-substituted (1*R*,3*R*,4*S*)-menthylamine, the diastereoselectivity is significantly improved, owing to the increased steric demand of the substituent on the stereocenter in vicinity to the oxime functionality. However, if the size of the substituent is further increased, the electrochemical conversion is significantly impaired.

## Supporting Information

File 1 Experimental details and ^1^H and ^13^C NMR spectra are provided.
