# Supplementary material for: Stereoselective cathodic synthesis of 8-substituted (1R,3R,4S)-menthylamines
Source: Beilstein J Org Chem. 2015 Feb 27;11:294–301. doi: 10.3762/bjoc.11.34 (PMC4362023; doi:10.3762/bjoc.11.34)
Supplement: File 1 — Experimental details and 1H and 13C NMR spectra are provided. [file Beilstein_J_Org_Chem-11-294-s001.pdf]

Supporting Information

for

Stereoselective cathodic synthesis of 8-substituted (1*R*,3*R*,4*S*)-menthylamines

Carolin Edinger, Jörn Kulisch, Siegfried R. Waldvogel*

Address: Institut für Organische Chemie, Johannes Gutenberg-Universität Mainz, Duesbergweg 10-14, 55128 Mainz, Germany

Email: Siegfried R. Waldvogel* - waldvogel@uni-mainz.de

*Corresponding author

Content

(1*R*)-8-Phenylmenthone (**6a**)....s3

(1*R*)-8-(3,5-Dimethoxyphenyl)menthone (**6b**)....s4

(1*R*)-8-(4-Biphenyl)menthone (**6c**)....s6

(1*R*,4*S*)-8-Phenylmenthone oxime (**7a**)....s8

(1*R*,4*S*)-8-(3,5-Dimethoxyphenyl)menthone oxime (**7b**)....s9

(1*R*,4*S*)-8-(4-Biphenyl)menthone oxime (**7c**)....s11

Preparation of the 8-substituted (1*R*,3*R*,4*S*)-menthylamines....s13

(−)-(1*R*,3*R*,4*S*)-8-Phenylmenthylamine (**8a**)....s14

(−)-(1*R*,3*R*,4*S*)-8-(3,5-Dimethoxyphenyl)menthylamine (**12a**)....s15

(−)-(1*R*,3*R*,4*S*)-8-Phenylmenthylammonium chloride (**8c**)....s17

(−)-(1*R*,3*R*,4*S*)-8-(3,5-Dimethoxyphenyl)menthylammonium chloride (**12c**)....s18

General remarks

All reagents were used in analytical grades. Solvents were desiccated if necessary by standard methods. *N*-Methyl-*N*,*N*,*N*-triethylammonium methylsulfate (MTES, **10**) and *N*,*N*,*N*´,*N*´-tetrabutyl-*N*,*N*´-diethyl-hexane-1,6-diammonium hydroxide (**11**) (7.8% in water, BQAOH) were used as supporting electrolytes and provided by the BASF SE, Germany. Column chromatography was performed on silica gel (particle size 63–200 µm, Merck, Darmstadt, Germany) by using mixtures of cyclohexane/*tert*-butyl methyl ether (MTBE) with triethyl amine or cyclohexane/ethyl acetate (EtOAc) as eluents. For thin-layer chromatography silica gel 60 sheets on glass (F_{254}, Merck, Darmstadt, Germany) were employed. Melting points were determined with a Melting Point Apparatus B-545 (Büchi, Flawil, Switzerland) and were uncorrected. Microanalysis was performed on a Vario MICRO cube (Elementar-Analysensysteme, Hanau, Germany). Gas Chromatography was performed with a HP 5890 Series II GC (Hewlett Packard, USA) using a RTX®200 Column (Restek, USA, length: 30 m, inner diameter: 0.25 mm, film: 0.5 µm, carrier gas: hydrogen) or with Shimadzu GC-2010 (Shimadzu, Japan), using a HP 5 (Agilent Technologies, USA; length: 30 m, inner diameter: 0.25 mm, film: 0.25 µm) or QP-2010 (Shimadzu, Japan) using a HP 1 (Agilent Technologies, USA; length: 30 m, inner diameter: 0.25 mm, film: 0.25 µm). ^{1}H NMR and ^{13}C NMR spectra were recorded at 25 °C by using a Bruker Avance DPX 300, DMX 300, DPX 400, DMX 500, DRX 500 or a Bruker AC 300 or AV II 400 instrument (Analytische Messtechnik, Karlsruhe, Germany). Chemical shifts (δ) referenced to TMS peak or residual solvent peak as internal standard are reported in parts per million (ppm) as s = singlet, d = doublet, t = triplet, m = multiplet with coupling constants in Hz. Mass spectra were obtained by using a MAT8200 or MAT95XL (Finnigan, Bremen, Germany) apparatus employing EI and by using micrOTOF-Q (Bruker-Daltonik, Bremen, Germany) apparatus or QTof Ultima 3 apparatus (Waters, Milford, Massachusetts) employing ESI (Thermo Finnigan, Bremen, Germany).

Preparation of 8-substituted menthone derivatives

(1*R*)-8-Phenylmenthone (6a)

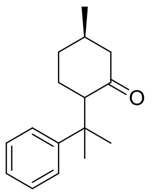

Under argon atmosphere magnesium turnings (4.20 g, 0.173 mol) in dry Et_2O (10 mL) were placed in a dried flask equipped with a dropping funnel and a condenser. The Grignard reagent was prepared by dropwise addition of bromobenzene (27.16 g, 0.173 mol) in anhyd Et_2O (60 mL). Subsequently, the mixture was refluxed for 30 min. Remaining Mg was removed by filtration under inert atmosphere and the filtrate was cooled to 0 °C before CuI (2.32 g, 0.012 mol) was added and the mixture was stirred for 30 min. At 0 °C (+)-pulegone (purity 92%, 15.00 g, 0.099 mol) in anhyd Et_2O (15 mL) was added dropwise. The reaction mixture was stirred overnight and allowed to warm up to r.t. Then H_2O (20 mL) was added (caution) while cooling, followed by addition of saturated aqueous ammonium chloride solution (80 mL) and 1 M HCl (20 mL). After extraction with MTBE (3 × 100 mL) the combined organic fractions were washed twice with saturated aqueous $Na_2S_2O_3$ and $NaHCO_3$ solutions (each 200 mL), dried over $MgSO_4$ and concentrated under reduced pressure. Purification by distillation in vacuum (10^{-3} mbar) yielded **6a** (15.87 g, 0.069 mol, 70%) as a slightly yellow oil [1].

Diastereomeric ratio 4:1 (NMR data cover both diastereomers)

^{1}H NMR (400 MHz, $CDCl_3$) δ = 0.91 (d, ^3J =7.1 Hz, 3H), 0.98 (d, ^3J = 6.6 Hz, 3H), 1.08-1.31 (m, 2H), 1.41, 1.43, 1.47 (s, 12H), 1.56-1.64 (m, 1H), 1.68-1.91 (m, 6H), 1.98-2.06 (m, 2H), 2.23-2.28 (m, 2H), 2.45-2.50 (m, 1H), 2.64-2.70 (m, 2H), 7.15-7.20 (m, 2H), 7.27-7.36 (m, 8H);

^{13}C NMR (100 MHz, $CDCl_3$) δ = 19.3, 22.3, 23.8, 24.0, 24.9, 26.5, 27.2, 29.0, 31.3, 32.2, 34.7, 36.3, 39.0, 39.5, 50.3, 52.4, 59.6, 59.7, 125.5, 125.6, 125.8, 125.9, 128.0, 149.4, 149.9, 211.2, 212.0;

Analytical data were consistent with literature [1].

(1*R*)-8-(3,5-Dimethoxyphenyl)menthone (6b)

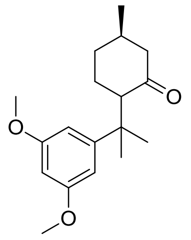

Under argon atmosphere magnesium turnings (1.12 g, 0.046 mol) in dry THF (10 mL) were placed in a dried flask equipped with a dropping funnel and a condenser. The Grignard reagent was prepared by dropwise addition of 1-bromo-3,5-dimethoxybenzene (10.00 g, 0.046 mol) in anhyd THF (60 mL). Subsequently, the mixture was refluxed for 30 min. Remaining Mg was removed by filtration under inert atmosphere and the filtrate was cooled to 0 °C before CuI (0.64 g, 0.003 mol) was added and the mixture was stirred for 30 min. At 0 °C (+)-pulegone (purity 92%, 4.07 g, 0.026 mol) in anhyd THF (6 mL) was added dropwise. The reaction mixture was stirred overnight and allowed to warm up to r.t. Then H_2O (20 mL) was added (caution) while cooling, followed by addition of saturated aqueous ammonium chloride solution (80 mL) and 1 M HCl (20 mL). After extraction with MTBE (3 × 100 mL) the combined organic fractions were washed twice with saturated aqueous $Na_2S_2O_3$ and $NaHCO_3$ solution (each 200 mL), dried over $MgSO_4$ and concentrated under reduced pressure. The residue was purified by column chromatography (eluent cyclohexane:EtOAc = 9:1) to give the product (5.11 g, 0.018 mol, 69% yield) as a viscous yellow oil [1].

Diastereomeric ratio 6:1; R_F(cyclohexane:EtOAc = 9:1): 0.33; (NMR data refer to major component (1*R*,4*S*)-8-(3,5-dimethoxyphenyl)menthone.

^{1}H NMR (300 MHz, $CDCl_3$) δ = 0.97 (d, ^3J =6.2 Hz, 3H), 1.22-1.28 (m, 1H), 1.37 (s, 3H), 1.39-1.41 (m, 1H), 1.43 (s, 3H), 1.72-1.84 (m, 3H), 2.02 (td, ^2J = 12.5 Hz, ^4J =1.3 Hz, 1H), 2.26 (ddd, ^2J = 12.4 Hz, ^3J = 4.0 Hz, ^4J = 2.1 Hz, 1H), 2.63 (ddd, ^2J = 12.9 Hz, ^3J =4.7 Hz, ^4J = 1.2 Hz, 1H), 3.79 (s, 6H), 6.30 (t, ^4J = 2.2 Hz, 1H), 6.49 (d, ^4J = 2.2 Hz, 2H);

^{13}C NMR (75 MHz, $CDCl_3$) δ = 21.5, 22.6, 26.0, 28.2, 33.9, 35.4, 38.6, 51.5, 54.4, 58.6, 95.8, 103.9, 151.9, 159.6, 210.4;

HRMS: *m*/*z* for $C_{18}H_{26}O_3$ calc: 290.1882, found: 290.1887;

6b: ^{1}H NMR (300 MHz, $CDCl_3$)

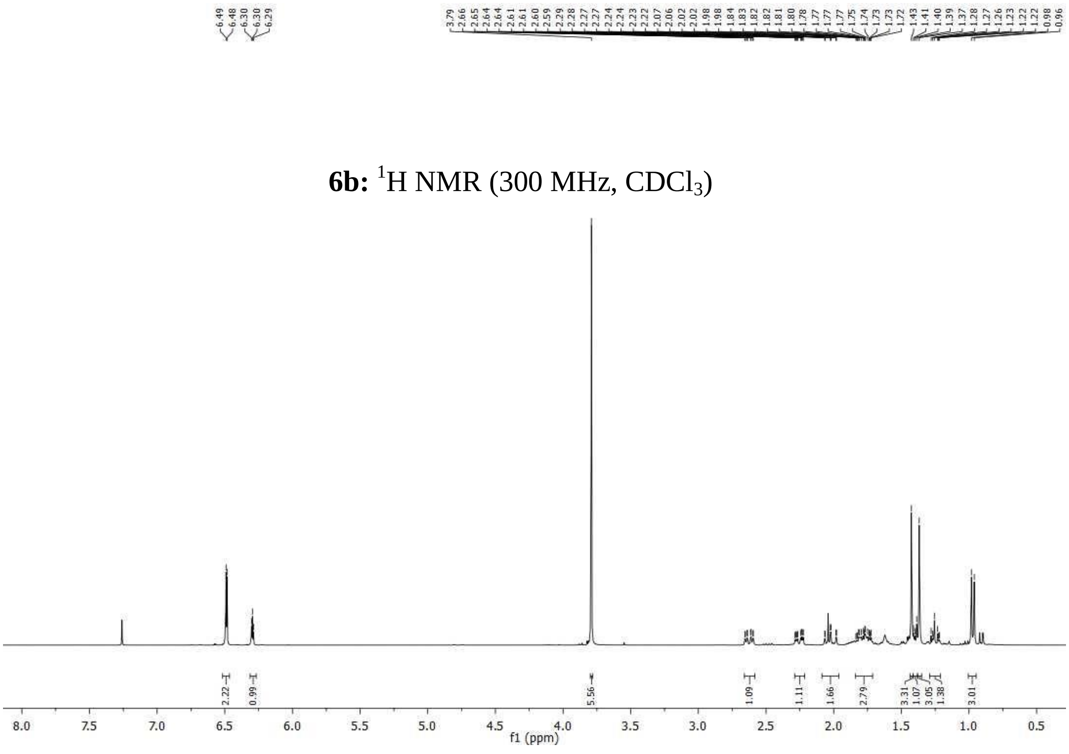

6b: ^{13}C NMR (75 MHz, $CDCl_3$)

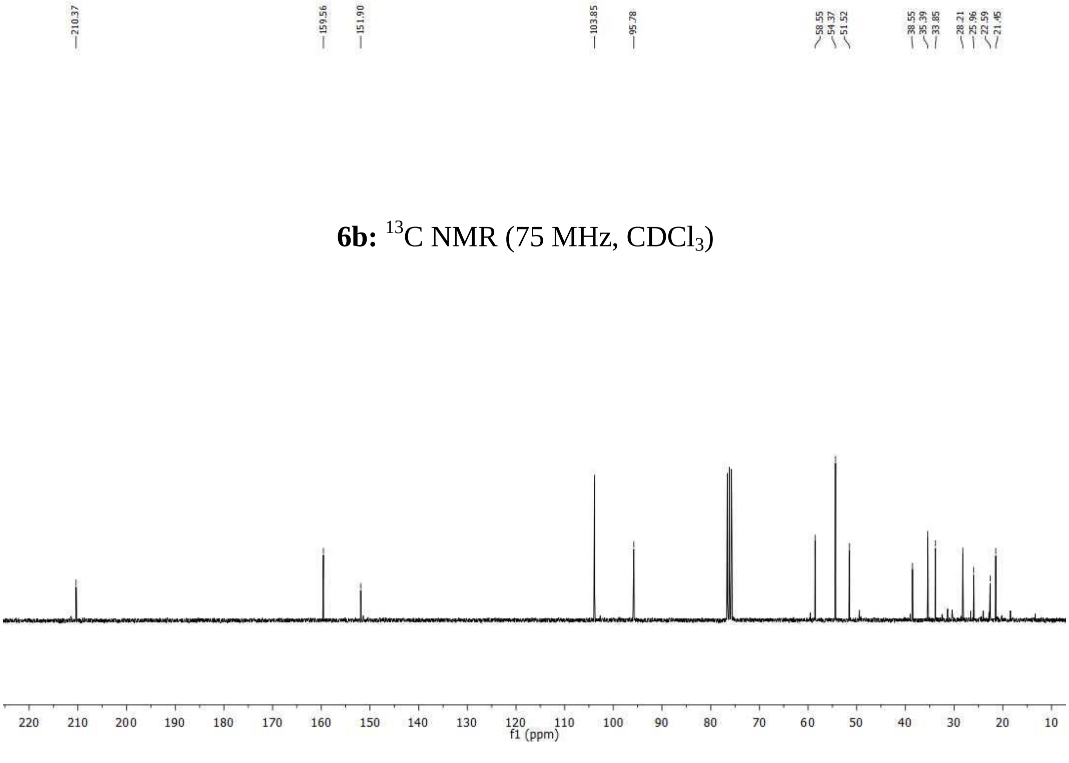

(1*R*)-8-(4-Biphenyl)menthone (6c)

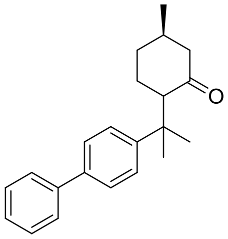

Under argon atmosphere magnesium turnings (2.18 g, 0.09 mol) in dry THF (11 mL) were placed in a dried flask equipped with a dropping funnel and a condenser. The Grignard reagent was prepared by dropwise addition of 1-bromo-diphenyl (17.48 g, 0.075 mol) in anhyd THF (60 mL). Subsequently, the mixture was refluxed for 30 min. Remaining Mg was removed by filtration under inert atmosphere and the filtrate was cooled to 0 °C before CuI (1.00 g, 0.005 mol) was added and the mixture was stirred for 30 min. At 0 °C (+)-pulegone (purity 92%, 9.13 g, 0.06 mol) in anhyd THF (15 mL) was added dropwise. The reaction mixture was stirred overnight and allowed to warm up to r.t. Then H_2O (20 mL) was added (caution) while cooling, followed by addition of saturated aqueous ammonium chloride solution (80 mL) and 1 M HCl (20 mL). After extraction with MTBE (3 × 100 mL) the combined organic fractions were washed twice with saturated aqueous $Na_2S_2O_3$ and $NaHCO_3$ solution (each 200 mL), dried over $MgSO_4$ and concentrated under reduced pressure. The residue was purified by short path distillation in vacuum (10^{-3} mbar) to yield **6c** (11.33 g, 0.037 mol, 62%) as a highly viscous red oil [1].

Diastereomeric ratio 3:1 (NMR data cover both diastereomeres).

^{1}H NMR (300 MHz, $CDCl_3$) δ = 0.92, 0,99 (d, ^3J =7.0 Hz, ^3J =6.6 Hz, 6H), 1.21-1.39 (m, 2H), 1.44, 1.46, 1.50, 1.53 (s, 12H), 1.61-1.93 (m, 8H), 1.96-2.12 (m, 2H), 2.27 (ddd, ^2J = 12.4 Hz, ^3J = 3.9 Hz, ^4J = 2.0 Hz, 2H), 2.50 (ddd, ^2J = 13.0 Hz, ^3J = 5.7 Hz, ^4J = 1.2 Hz, 2H), 2.71 (ddd, ^2J = 13.1 Hz, ^3J = 4.6 Hz, ^4J = 1.2 Hz, 2H), 7.28-7.37 (m, 2H), 7.37-7.48 (m, 8H), 7.50-7.63 (m, 8H);

^{13}C NMR (75 MHz, $CDCl_3$) δ = 19.4, 22.5, 24.3, 25.1, 26.6, 27.2, 29.2, 31.4, 32.4, 34.9, 36.4, 39.0, 39.5, 50.5, 52.5, 59.7, 126.4, 126.8, 127.1, 128.8, 138.3, 141.0, 149.2, 211.5;

HRMS: *m/z* for $C_{22}H_{27}O$ calc: 307.2062, found: 307.2064;

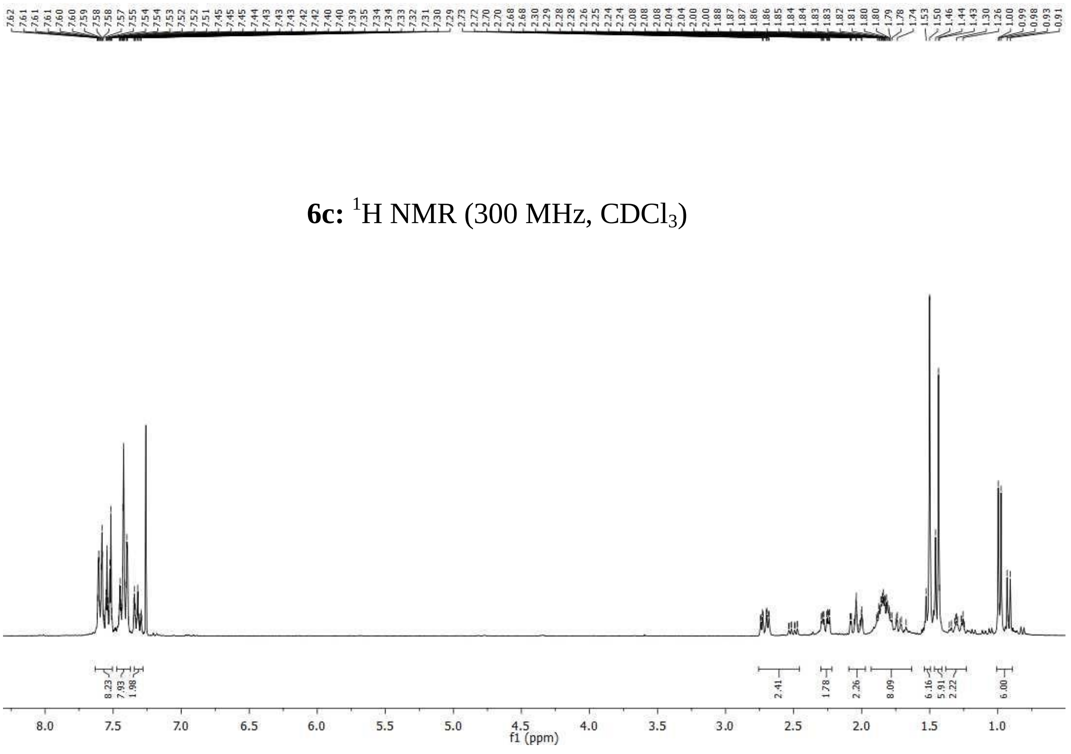

6c: ^{1}H NMR (300 MHz, $CDCl_3$)

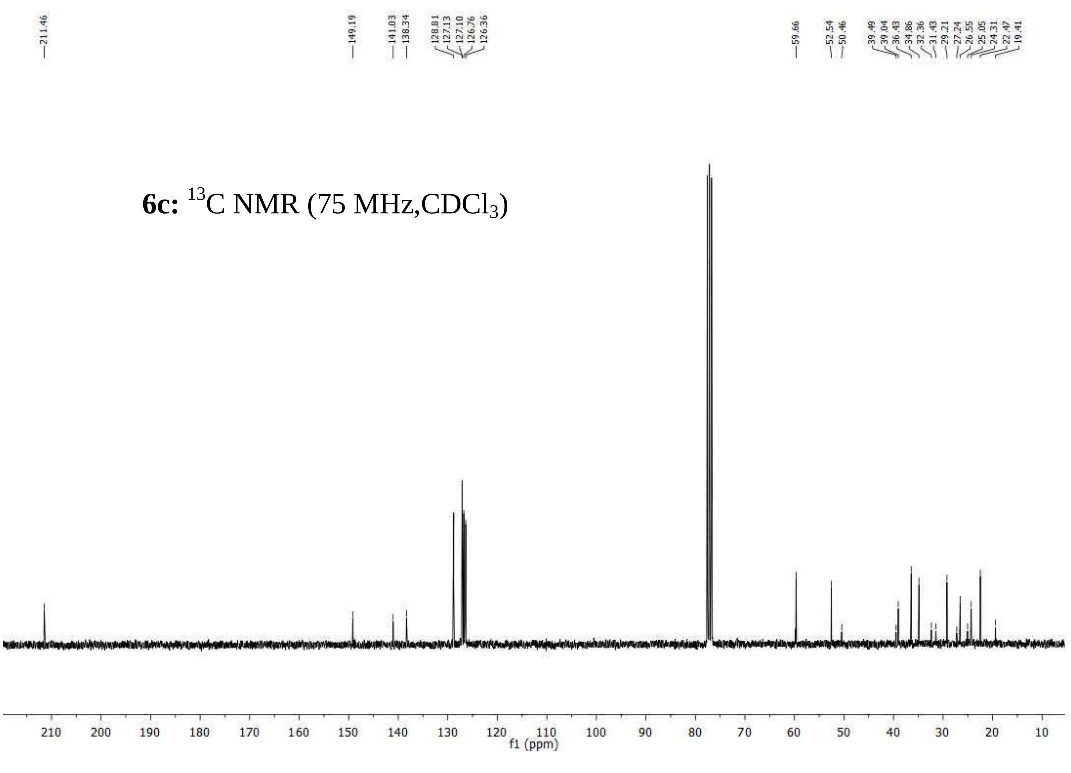

6c: ^{13}C NMR (75 MHz,$CDCl_3$)

Preparation of 8-substituted (1*R*,4*S*)-oxime derivatives

General protocol: Preparation of the 8-substituted (1*R*,4*S*)-menthone oximes (7)

Menthone derivatives (0.02 mol) were dissolved in EtOH (100 mL). Hydroxylammonium chloride (0.18 mol, 12.51 g), NaOH (0.36 mol, 7.40 g), and water (100 mL) were added and the suspension was stirred at 25 °C until completion of reaction. EtOH was removed under reduced pressure. The mixture was diluted with water (50 mL) and extracted with MTBE (3 × 50 mL). The combined organic fractions were washed with brine, dried over $MgSO_4$ and liberated from volatile components on a rotary evaporator. The crude products were purified by crystallization or column chromatography [1].

(1*R*,4*S*)-8-Phenylmenthone oxime (7a)

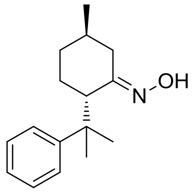

Yield: 4.17 g (0.017 mol, 85%) slightly yellow solid; purification by column chromatography on silica gel; m.p. 61-63 °C; R_f(cyclohexane:EtOAc = 9:1): 0.40;

^{1}H NMR (400 MHz, $CDCl_3$) δ = 0.96 (d, ^{3}J = 6.0 Hz, 3H), 0.98-1.03 (m, 1H), 1.32-1.39 (m, 1H), 1.43 (s, 3H), 1.49 (s, 3H), 1.60-1.73 (m, 4H), 2.52-2.56 (m, 1H), 2.99-3.06 (m, 1H), 7.16-7.19 (m, 1H), 7.28-7.38 (m, 4H);

^{13}C NMR (100 MHz, $CDCl_3$) δ = 22.0, 25.0, 26.9, 27.9, 32.5, 32.7, 33.8, 40.1, 52.3, 125.2, 125.7, 127.7, 149.9, 160.8;

Analytical data were consistent with the literature [1].

(1*R*,4*S*)-8-(3,5-Dimethoxyphenyl)menthone oxime (7b)

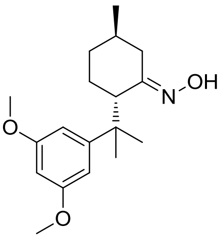

Yield: 4.99 g (0.016 mol, 81%) viscous yellow oil; purification by column chromatography on silica gel; R_F(cyclohexane:EtOAc = 4:1) = 0.50;

^{1}H NMR (400 MHz, $CDCl_3$) δ = 0.93 (d, ^3J = 6.1 Hz, 3H), 1.10-1.28 (m, 1H), 1.36 (s, 3H), 1.39 (s, 3H), 1.41-1.47 (m, 2H), 1.50-1.73 (m, 3H), 2.59 (dd, ^3J = 6.7 Hz, ^4J = 3.8 Hz, 1H), 3.20 (dd, ^3J = 6.8 Hz, ^4J = 3.8 Hz, 1H), 3.97 (s, 6H), 6.32 (t, ^4J = 2.2 Hz, 1H), 6.54 (d, ^4J = 2.2 Hz, 2H);

^{13}C NMR (100 MHz, $CDCl_3$) δ = 22.2, 26.3, 26.5, 30.0, 31.3, 31.5, 31.6, 41.8, 49.4, 55.4, 97.2, 105.6, 151.6, 160.5, 161.5;

HRMS: *m*/*z* for $C_{18}H_{27}NO_3$ calc: 305.1991, found: 305.2001.

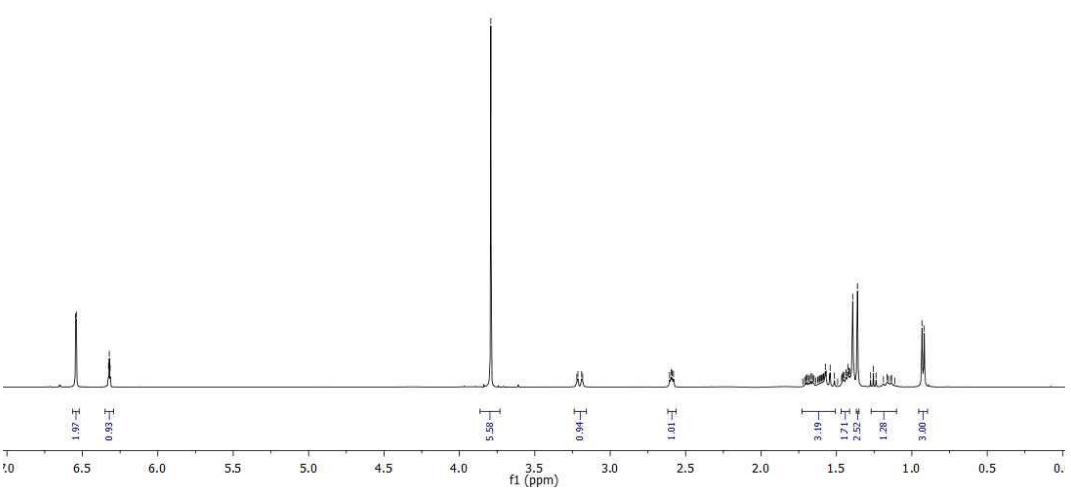

7b: ^{1}H NMR (400 MHz, $CDCl_3$)

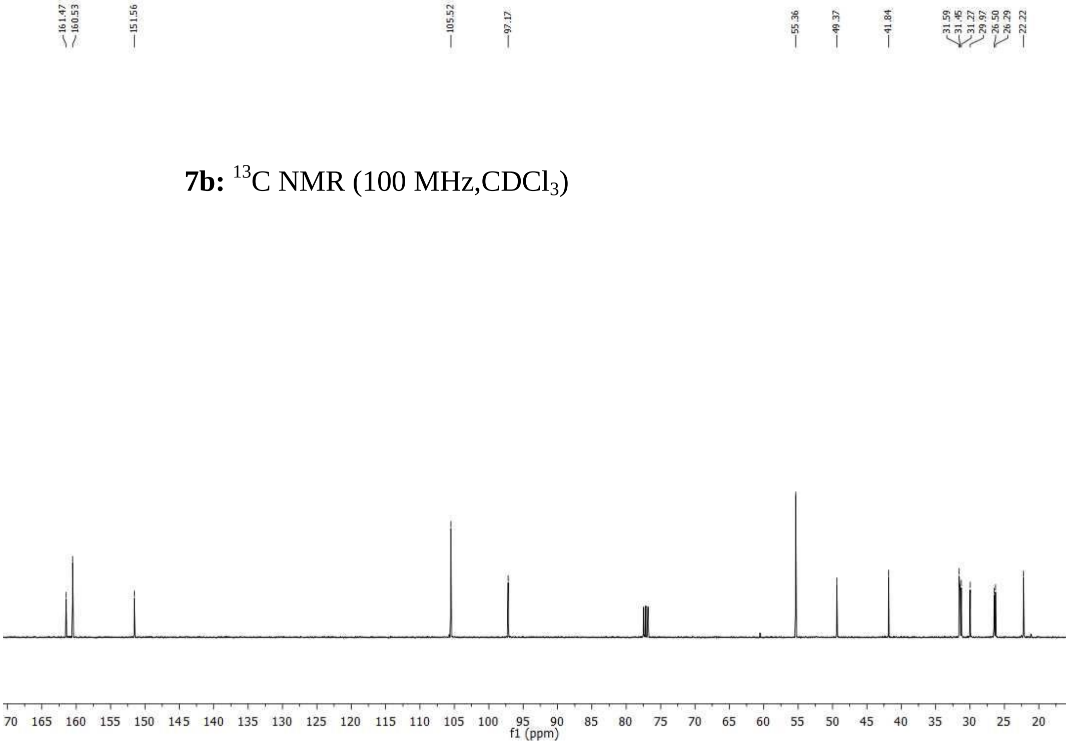

7b: ^{13}C NMR (100 MHz,$CDCl_3$)

(1*R*,4*S*)-8-(4-Biphenyl)menthone oxime (7c)

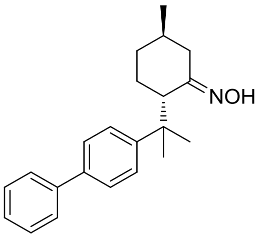

Yield: 4.38 g (0.014 mol, 68%) colorless solid; m.p. 168-171 °C; purification by column chromatography on silica gel; R_F(cyclohexane:EtOAc = 5:1) = 0.43;

^{1}H NMR (300 MHz, DMSO-d_6) δ = 0.95 (d, ^3J = 6.4 Hz, 3H), 1.02-1.12 (m, 1H), 1.27-1.47 (m, 2H), 1.47 (s, 3H), 1.54 (s, 3H), 1.57-1.61 (m, 1H), 1.64-1.74 (m, 2H), 2.62 (dd, ^2J = 11.9 Hz, ^3J = 3.8 Hz, 1H), 3.31 (ddd, ^2J = 12.7 Hz, ^3J = 4.2 Hz, ^4J = 1.8 Hz, 1H), 7.31-7.37 (m, 1H), 7.43-7.51 (m, 4H), 7.52-7.61 (m, 2H), 7.65-7.68 (m, 2H), 9.39 (s, OH);

^{13}C NMR (75 MHz, DMSO-d_6) δ = 22.6, 25.3, 27.3, 29.1, 34.0, 34.2, 35.5, 40.7, 53.3, 127.0, 127.3, 127.4, 127.8, 129.6, 138.5, 141.6, 151.1, 159.3;

HRMS: *m*/*z* for $C_{22}H_{28}NO$ calc: 322.2171, found: 322.2168.

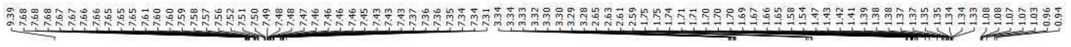

7c: ^{1}H NMR (300 MHz, DMSO-d_6)

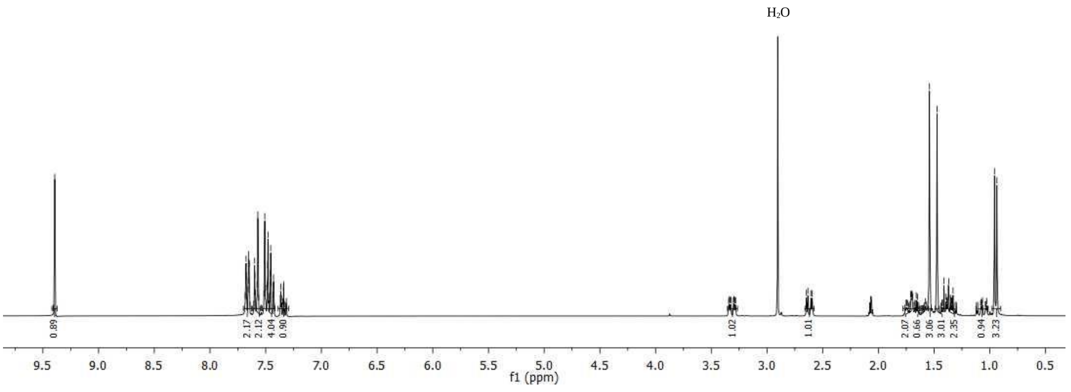

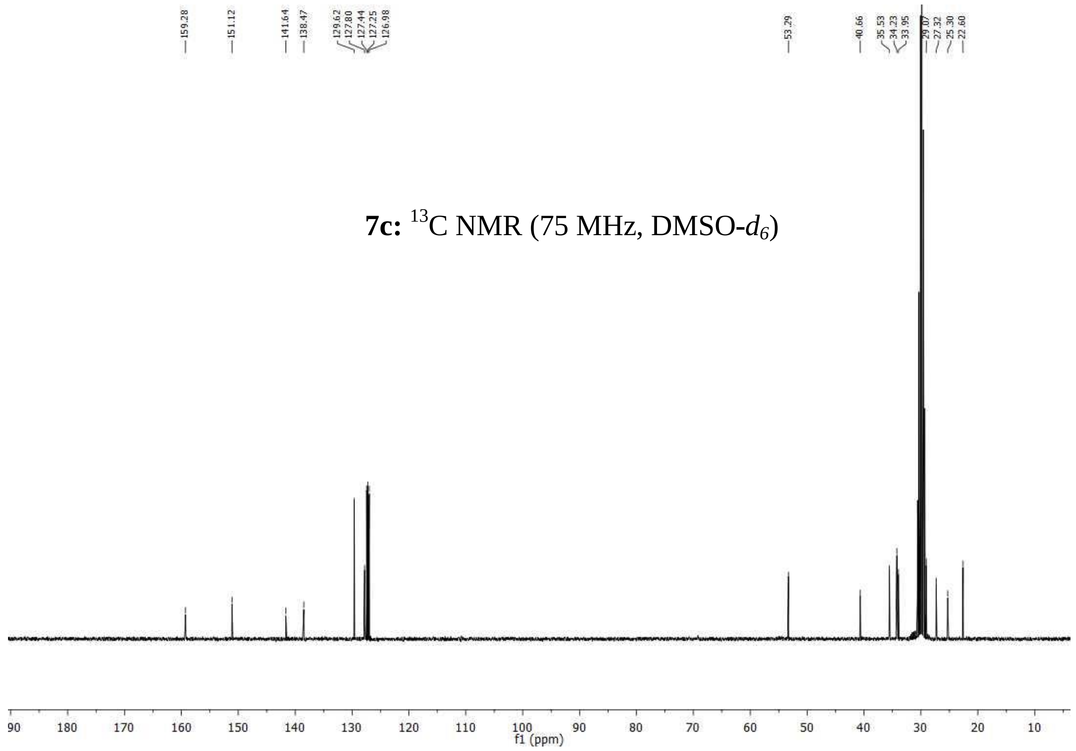

7c: ^{13}C NMR (75 MHz, DMSO-d_6)

Preparation of the 8-substituted (1*R*,3*R*,4*S*)-menthylamines

General protocol for electroreductive amine formation

A solution of oximes (2.06–3.94 mmol) and electrolyte (50.0 g) was transferred into the cathodic compartment of a divided electrolysis cell separated by Nafion™ 324 membrane. The anodic compartment was filled with a solution of sulfuric acid (1.0 g, 0.010 mol) in methanol (49.0 g). By using a platinum anode (5 cm^2) and a lead cathode (7 cm^2), at 20 °C galvanostatic electrolysis with a current density of 12.5 mA/cm^2 was performed. After completion (approx. 10 F per mol oxime) the electrolysis mixture was transferred with methanol (50 mL). The reaction mixture was diluted with water (50 mL), brought to pH ≤1 by means of concentrated sulfuric acid and washed with cyclohexane (3 × 50 mL). The aqueous phase is subsequently brought to pH ≥12 by means of potassium hydroxide solution (50%) and extracted with MTBE (3 × 50 mL). These combined organic fractions were dried over powdered calcium oxide and the solvent was removed on a rotary evaporator. Further purification was accomplished by column chromatography or distillation [2].

Experimental set-up:

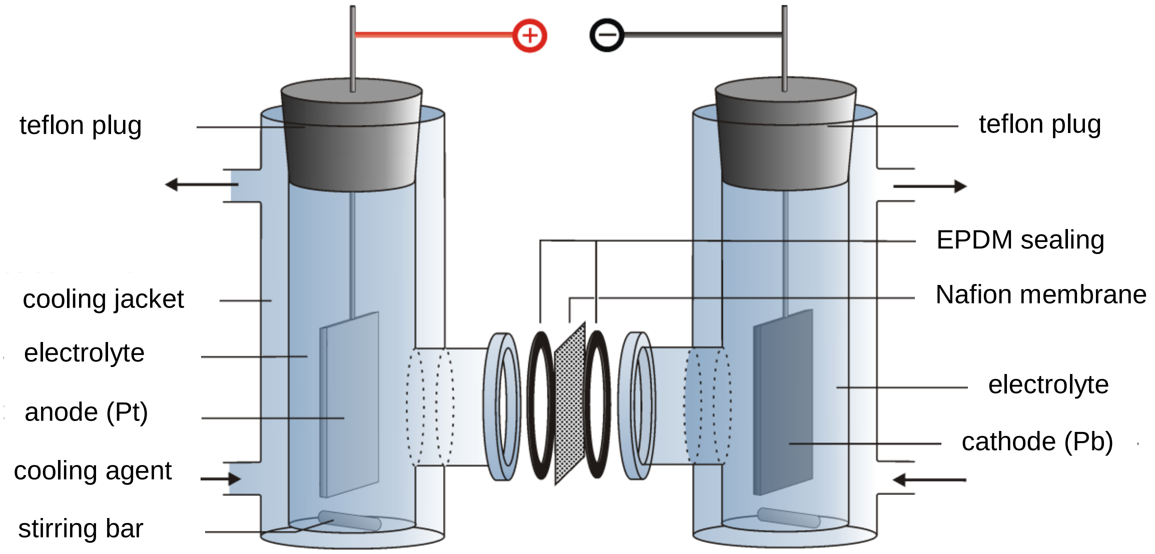

Figure 1: Experimental set-up for the electrochemical synthesis of 8-substituted (1*R*,3*R*,4*S*)-menthylamines [2].

(−)-(1*R*,3*R*,4*S*)-8-Phenylmenthylamine (8a)

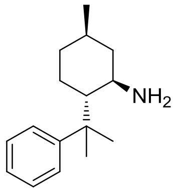

Yield: 0.46 g (2 mmol, 93%, diastereomeric ratio 8:1) slightly yellow oil; electrolyte: triethylmethylammonium methylsulfate (0.25 g, 0.001 mol) sulfuric acid (1.00 g, 0.010 mol) in methanol (48.75 g); purification by distillation; b.p. 86 °C (1.2 × 10^{-2} mbar); $[\alpha]_D^{20}$ = −26.9° (c = 0.8 in $CHCl_3$); t_R = 9.8 min (HP 5890 Series II GC, RTX®200, method: 50 °C, rate 15 °C/min up to 290 °C, 290 °C for 8 min);

^{1}H NMR (400 MHz, $CDCl_3$) δ = 0.82-0.73 (m, 1H), 0.85 (d, ^3J = 6.5 Hz, 3H), 0.84-0.92 (m, 1H), 1.08 (dddd, ^2J = 13.1 Hz, ^3J = 12.2 Hz, ^3J = 3.3 Hz, 1H), 1.19 (s, 3H), 1.36 (s, 3H), 1.32-1.43 (m, 1H), 1.59-1.71 (m, 3H), 1.80 (dddd, ^2J = 13.1 Hz, ^3J = 3.3 Hz, 1H), 2.58-2.64 (m, 1H), 7.11-7.15 (m, 1H), 7.25-7.29 (m, 2H), 7.35-7.37 (m, 2H);

^{13}C NMR (100 MHz, $CDCl_3$) δ = 22.4, 22.8, 27.2, 30.8, 32.2, 35.5, 40.1, 46.8, 53.4, 54.4, 125.5, 128.5, 153.1;

Analytical data were consistent with the literature [1].

(−)-(1*R*,3*R*,4*S*)-8-(3,5-Dimethoxyphenyl)menthylamine (12a)

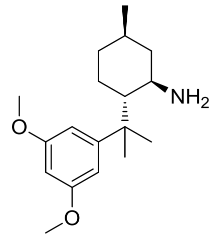

Yield: 1.03 g (4 mmol, 89%, diastereomeric ratio 9:1) slightly yellow oil; electrolyte: BQAOH (7.8% in water, 6.41 g) sulfuric acid (1.00 g, 0.010 mol) in methanol (41.59 g); purification by distillation; b.p.: 120-125°C (6×10^{-3} mbar); t_R = 12.2 min (HP 5890 Series II GC, RTX®200, method: 50 °C, rate 15 °C/min up to 290 °C, 290 °C for 8 min); $[\alpha]_D^{20} = -30.4°$ (c = 1.1 in $CHCl_3$);

^{1}H NMR (400 MHz, $CDCl_3$) δ = 0.80-0.83 (m, 1H), 0.86 (d, ^3J = 6.5 Hz, 3H), 0.90-1.17 (m, 2H), 1.18 (s, 3H), 1.33 (s, 3H), 1.35-1.56 (m, 2H), 1.60-1.74 (m, 2H), 1.72-1.87 (m, 2H), 1.90 (bs, 2H), 3.79 (s, 6H), 6.28 (t, ^4J = 2.2 Hz, 1H), 6.52 (d, ^4J = 2.2 Hz, 2H);

^{13}C NMR (100 MHz, $CDCl_3$) δ = 22.3, 23.1, 27.1, 30.5, 32.1, 35.4, 40.3, 46.6, 53.4, 54.4, 55.3, 96.6, 104.4, 155.8, 160.7;

HRMS: *m/z* for $C_{18}H_{29}NO_2$ calc: 291.2198, found: 291.2197;

Elem. Anal. calc (%): C 74.18, H 10.03, N 4.81, found (%): C 74.16, H 10.20, N 4.41.

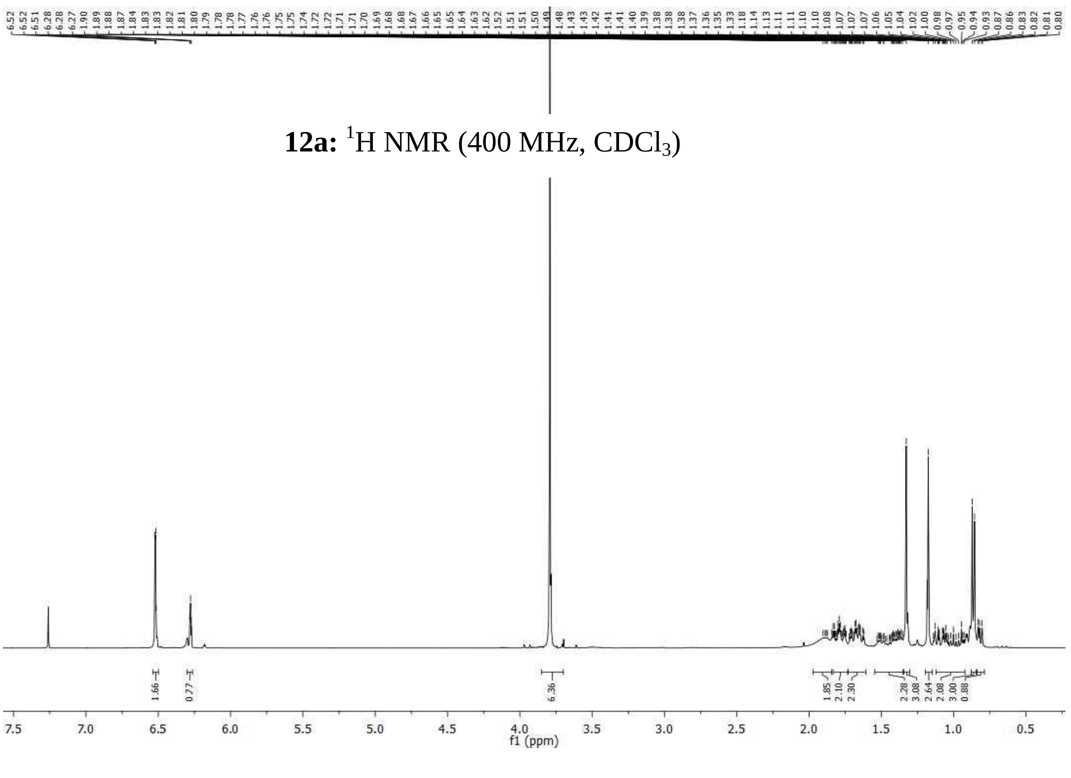

12a: ^{1}H NMR (400 MHz, $CDCl_3$)

12a: ^{13}C NMR (100 MHz, $CDCl_3$)

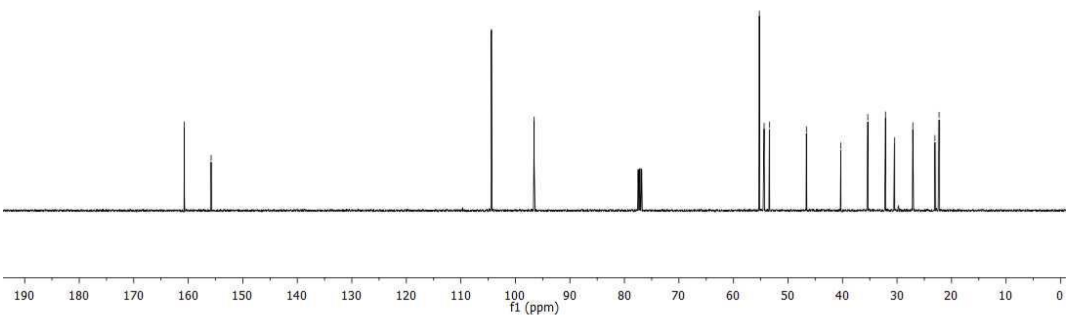

Preparation of the 8-substituted (−)-(1*R*,3*R*,4*S*)-menthylammonium chlorides

Preparation of the 8-substituted (−)-(1*R*,3*R*,4*S*)- menthylammonium chlorides: General Procedure

The menthylamines (11-20 mmol) were dissolved in Et_2O (50 mL) and treated at 0 °C with HCl–Et_2O (10 equiv.). The resulting solids were crystallized from 1,4-dioxane (**8c**) or CH_2Cl_2/hexane (**12c**), respectively yielding the products as colorless crystals [2].

(−)-(1*R*,3*R*,4*S*)-8-Phenylmenthylammonium chloride (8c)

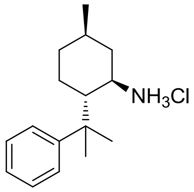

Yield: 1.31 g (5 mmol, 73%) colorless solid; m.p. 198 °C (sublimation); $[\alpha]_D^{20} = -18.3°$ (c = 1.02 in MeOH);

^{1}H NMR (400 MHz, DMSO-d$_6$) δ = 0.64 (ddd, ^2J = 12.6 Hz, ^3J = 12.6 Hz, ^3J = 3.1 Hz, 1H), 0.83 (d, ^3J = 6.4 Hz, 3H), 0.93 (ddd, ^2J = 15.5 Hz, ^3J = 13.2 Hz, ^3J = 3.0 Hz, 1H), 1.06-1.15 (m, 2H), 1.28 (s, 3H,), 1.44 (s, 3H), 1.34-1.45 (m, 2H), 1.92-1.98 (m, 1H), 2.07-2.10 (m, 1H), 3.08-3.10 (m, 1H), 7.15-7.20 (m, 1H), 7.31-7.32 (m, 4H), 7.94 (bs, 3H);

^{13}C NMR (100 MHz, DMSO-d$_6$) δ = 21.6, 22.7, 27.1, 28.8, 30.7, 33.7, 40.3, 41.0, 49.4, 51.6, 125.3, 125.6, 128.3, 150.6;

Analytical data were consistent with the literature [1].

(−)-(1*R*,3*R*,4*S*)-8-(3,5-Dimethoxyphenyl)menthylammonium chloride (12c)

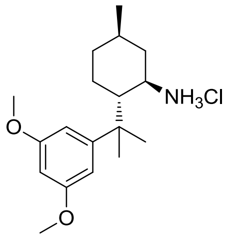

Yield: 3.20 g (10 mmol, 88%) colorless solid; m.p.: 193.7 °C; $[\alpha]_D^{20} = -29.7°$ (c = 1.1 in $CHCl_3$);

^{1}H NMR (500 MHz, DMSO-*d6*) δ = 0.70 (ddd, ^2J = 25.0 Hz, ^3J = 12.6 Hz, ^3J = 3.0 Hz, 1H), 0.84 (d, ^3J = 6.5 Hz, 3H), 1.03 (m, 2H), 1.21 (s, 3H), 1.26-1.32 (m, 2H), 1.38 (s, 3H), 1.50 (d, ^3J = 12.9 Hz, 1H), 1.86-2.01 (m, 1H), 2.06 (d, ^3J = 12.3 Hz, 1H), 3.74 (s, 6H),), 6.34 (t, ^4J = 2.2 Hz, 1H), 6.45 (d, ^4J = 2.2 Hz, 2H), 7.80 (bs, 3H);

^{13}C NMR (125 MHz, $CDCl_3$) δ = 21.7, 24.0, 26.9, 27.5, 30.8, 33.8, 40.9, 49.3, 51.7, 55.1, 97.1, 104.1, 153.0, 160.4;

HRMS: *m*/*z* for $C_{18}H_{30}NO_2^+$ calc: 292.2271, found: 292.2269.

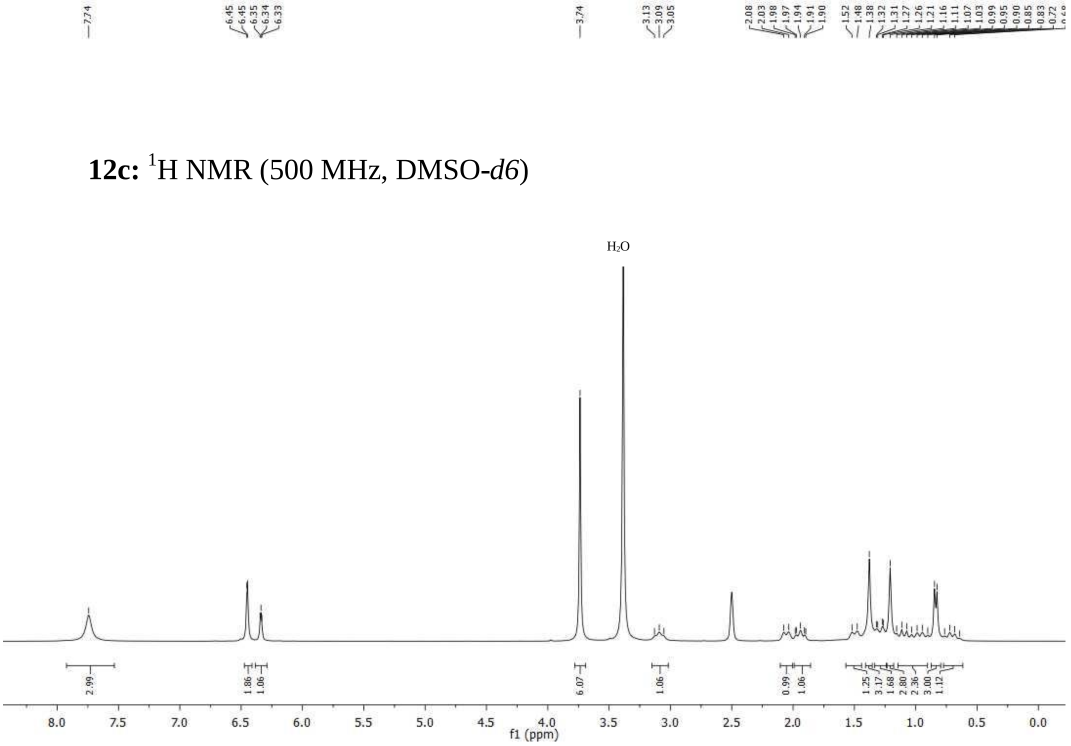

12c: ^{13}C NMR (500 MHz, DMSO-*d6*)

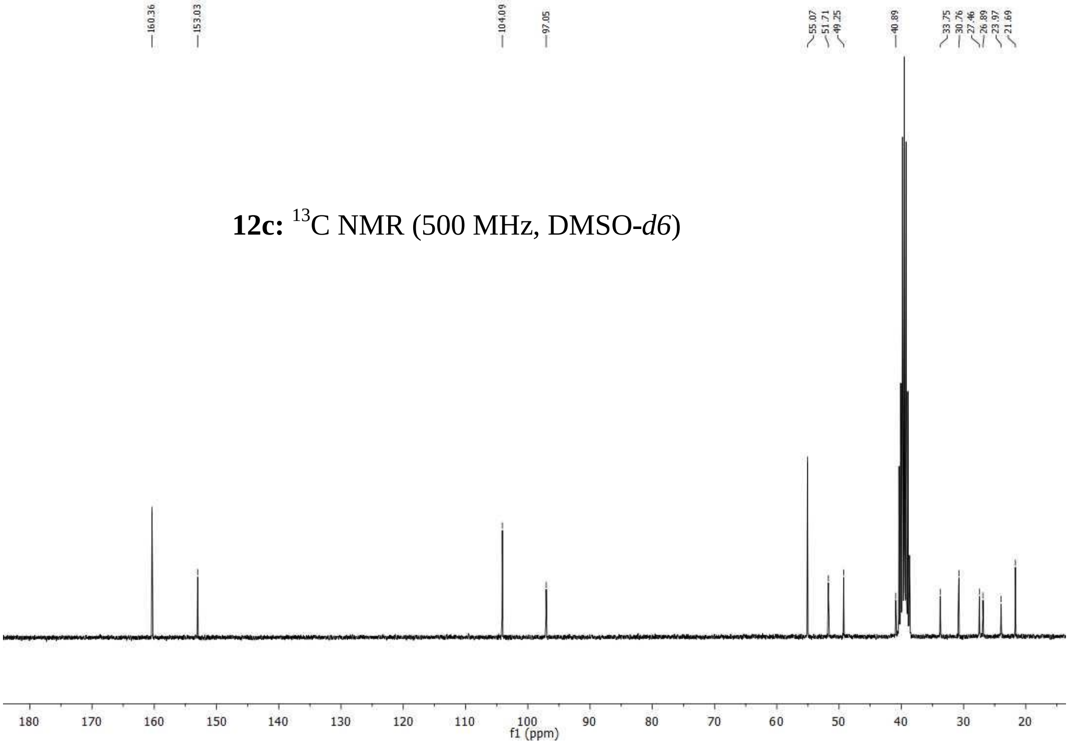

References

1. Schopohl, M. C.; Bergander, K.; Kataeva, O.; Fröhlich, R.; Waldvogel, S. R. *Synthesis*. **2003**, 2689–2694.

2. Kulisch, J.; Nieger, M.; Stecker, F.; Fischer, A.; Waldvogel, S. R. *Angew. Chem.* **2011**, *123*, 5678–5682; *Angew. Chem. Int. Ed.* **2011**, *50*, 5564–5567.
